# Ribociclib-Induced Cutaneous Adverse Events in Metastatic HR+/HER2− Breast Cancer: Incidence, Multidisciplinary Management, and Prognostic Implication

**DOI:** 10.1093/oncolo/oyae004

**Published:** 2024-01-18

**Authors:** Riccardo Giovanni Borroni, Michela Bartolini, Mariangela Gaudio, Flavia Jacobs, Chiara Benvenuti, Riccardo Gerosa, Paola Tiberio, Sofia Ada Assunta Maria Manara, Alessandra Solferino, Armando Santoro, Rita De Sanctis

**Affiliations:** Department of Biomedical Sciences, Humanitas University, Pieve Emanuele (MI), Italy; Dermatology Unit, IRCCS Humanitas Research Hospital, Rozzano (MI), Italy; Department of Biomedical Sciences, Humanitas University, Pieve Emanuele (MI), Italy; Medical Oncology and Hematology Unit, IRCCS Humanitas Research Hospital, Rozzano (MI), Italy; Department of Biomedical Sciences, Humanitas University, Pieve Emanuele (MI), Italy; Medical Oncology and Hematology Unit, IRCCS Humanitas Research Hospital, Rozzano (MI), Italy; Department of Biomedical Sciences, Humanitas University, Pieve Emanuele (MI), Italy; Medical Oncology and Hematology Unit, IRCCS Humanitas Research Hospital, Rozzano (MI), Italy; Department of Biomedical Sciences, Humanitas University, Pieve Emanuele (MI), Italy; Medical Oncology and Hematology Unit, IRCCS Humanitas Research Hospital, Rozzano (MI), Italy; Department of Biomedical Sciences, Humanitas University, Pieve Emanuele (MI), Italy; Medical Oncology and Hematology Unit, IRCCS Humanitas Research Hospital, Rozzano (MI), Italy; Medical Oncology and Hematology Unit, IRCCS Humanitas Research Hospital, Rozzano (MI), Italy; Pathology Unit, IRCCS Humanitas Research Hospital, Rozzano (MI), Italy; Medical Oncology and Hematology Unit, IRCCS Humanitas Research Hospital, Rozzano (MI), Italy; Department of Biomedical Sciences, Humanitas University, Pieve Emanuele (MI), Italy; Medical Oncology and Hematology Unit, IRCCS Humanitas Research Hospital, Rozzano (MI), Italy; Department of Biomedical Sciences, Humanitas University, Pieve Emanuele (MI), Italy; Medical Oncology and Hematology Unit, IRCCS Humanitas Research Hospital, Rozzano (MI), Italy

**Keywords:** ribociclib, eczematous dermatitis, progression-free survival, metastatic breast cancer, endocrine therapy

## Abstract

**Background:**

Ribociclib is approved for hormone receptor positive (HR+), human epidermal growth factor receptor 2 negative (HER2−) advanced breast cancer (ABC) treatment, in combination with endocrine therapy. Hematological, hepatic, and cardiac adverse events (AEs) emerged from pivotal trials, but little is known about cutaneous adverse events (CAEs).

**Patients and Methods:**

We report data from a retrospective cohort study of all patients with HR+/HER2− ABC treated with ribociclib at Humanitas Cancer Center between June 2017 and December 2022. We recorded clinical-pathological data, the incidence, and treatment of ribociclib-related CAEs. These were evaluated according to the NCI-CTCAE v5.0 classification. Progression-free survival (PFS) was estimated by Kaplan-Meier method and the log-rank test was used to analyze differences between groups.

**Results:**

Thirteen of 91 patients (14.3%) experienced treatment-related CAEs (mean time to the occurrence: 3.9 months). The most frequent CAEs were eczematous dermatitis (53.8%) and maculo-papular reaction (15.4%). Itch was reported by all 13 patients. The grade was G3 in 8 cases, G2 in 4, and G1 in 1. An integrated approach based on ribociclib dose modulation and dermatological interventions (oral antihistamine, moisturized cream, topical, and/or systemic steroids) could prevent ribociclib discontinuation in most patients. At a median follow-up of 20 months, the median PFS was 13 months (range, 1-66) with a better PFS curves for patients experiencing CAEs (*P* = .04).

**Conclusion:**

We mapped frequency and types of ribociclib-induced CAEs. An interdisciplinary management of CAEs incorporated into routine care may reduce the rate of drug discontinuation thus potentially contributing to better long-term outcomes.

## Teaser

This study investigated the incidence and the characteristics of dermatological adverse events related to ribociclib and their impact on safety and efficacy of the treatment.

**Implications for Practice**

The study provides crucial insights into the incidence of ribociclib-induced cutaneous adverse events (CAEs) in a real-world population and describes the most common CAEs, such as eczematous dermatitis and maculopapular reaction. Interdisciplinary management of these adverse events (AEs), including ribociclib dose modulation and dermatological interventions, was found to prevent permanent drug discontinuation in the majority of patients. These results underscore the importance of a multidisciplinary approach to manage AEs associated with ribociclib in patients with HR+/HER2− advanced breast cancer. By incorporating this approach into routine care, clinicians can enhance treatment adherence and optimize patient outcomes in this specific patient population.

## Introduction

Cyclin-dependent kinase 4 and 6 inhibitors (CDK4/6i) have changed the therapeutic paradigm of hormone receptor (HR) positive, human epidermal growth factor receptor 2 (HER2) negative advanced breast cancer (ABC). The combination of CDK4/6i and endocrine therapy (ET) significantly improved the outcome of patients with HR+/HER2− ABC in terms of response rate and survival.^1-6^ This led to their approval in combination with a nonsteroidal aromatase inhibitor (NSAI, for endocrine-sensitive) or fulvestrant (mainly for endocrine-resistant). Currently, 3 different CDK4/6i (palbociclib, ribociclib, and abemaciclib) are used in clinical practice based on the clinical characteristics of patients, such as comorbidities and concomitant medications, together with their different toxicity profiles. More specifically, the updated results of the pivotal trials showed a significant improvement in overall survival (OS) of ribociclib plus ET with respect to ET alone, with a median OS of 58.7 months with ribociclib versus 48.0 months with placebo.^7^ In addition, extended follow-up in MONALEESA-3 reported a significantly improved median OS of 53.7 months with ribociclib plus fulvestrant versus 41.5 months with placebo plus fulvestrant.^8^ Similarly, in the endocrine-resistant disease, MONARCH-2 trial showed a statistically significant improvement in OS with abemaciclib plus fulvestrant versus ET alone (46.7 vs 37.3 months).^9^ To date, no mature OS data are available for abemaciclib plus NSAI (MONARCH-3),^3^ while palbociclib plus ET led to a numerically but not statistically significant improvement in median OS.^10,11^ However, after over 6 years of median follow-up, PALOMA-3 trial showed that patients with HR+/HER2− ABC who progressed on prior ET reported a clinically meaningful improvement in OS of 6.8 months with palbociclib plus fulvestrant versus placebo plus fulvestrant.^12^

Several studies on these 3 approved CDK4/6i demonstrated that their toxicity profile can be easily managed with supportive care measures, dose reduction, and/or temporary drug interruptions.^1-6,13-15^ Hematologic side effects, such as neutropenia, anemia, and thrombocytopenia, are the most commonly experienced by patients receiving CDK4/6i-based therapy. Alongside the hematological toxicities (70%), some distinct side effects are associated with each CDK4/6i. For instance, abemaciclib has been associated with increased diarrhea (87%) and fatigue (43%), whereas ribociclib has been linked to hepatotoxicity (12%) and early reversible, concentration-dependent prolongation of the QT interval (11%).^16,17^

A variety of cutaneous adverse events (CAEs) has been reported in both pivotal and real-world studies during the treatment with CDK4/6i and mainly included alopecia, CAEs, and pruritus.^1-6,18,19^ These side effects are particularly relevant, not only because of the potential impairment of patients’ health-related quality of life, but also because they can lead to treatment discontinuation. Overall, alopecia occurred in 23% of patients treated with CDK4/6i + ET compared with 9.6% of patients treated with ET alone, with a higher risk with abemaciclib. The incidence of CAEs and pruritus was approximately 15%-20% of patients treated with CDK4/6i + ET, with very few grade ≥3 (<1%).^20,21^ To date, there is little data on the characteristics of these skin manifestations and no information on their potential impact on the efficacy and outcome of the treatments.^18-20,22^

MONALEESA-2, MONALEESA-3, and MONALEESA-7 reported CAEs as one of the observed side effects. In detail, MONALEESA-2 reported 74 (22%) cases of CAEs and maculopapular reactions of any grade (G), MONALEESA-3 referred 96 (20%) cases of pruritus and 89 (18%) cases of CAEs while MONALEESA-7 reported 43 (13%), 31 (9%), and 27 (8%) cases of CAEs, pruritus, and dry skin, respectively.^2,6,23^ While pivotal trials only reported unspecific cases of mild CAEs, a wide range of dermatological manifestations, including severe ones, have been described with ribociclib in various case reports and series in literature.^24-26^

Therefore, to better investigate the incidence and the characteristics of dermatological adverse events (AEs) related to ribociclib and their impact on safety and efficacy of the treatment, we designed a retrospective observational cohort study enrolling patients with HR+/HER2− ABC who received ribociclib + ET in a real-world setting. In addition, we searched for possible predictive factors of CAEs related to ribociclib. Finally, we tested whether CAEs could work as a positive prognostic factor, consistently with recent studies.^27^

## Methods

### Study Design and Population

We conducted a retrospective observational study of all consecutive patients affected by HR+/HER2− ABC treated with ribociclib + ET from June 2017 to December 2022 at our Institution, IRCCS Humanitas Research Hospital (Milan). Those patients who decided to continue the therapy at another hospital were excluded from the analysis. For each patient, we reviewed electronic medical records and recorded demographic characteristics (age at diagnosis), baseline clinical-pathological characteristics (comorbidity, personal history of allergies and/or previous skin disease, Eastern Cooperative Oncology Group [ECOG] Performance Status, American Joint Committee on Cancer stage, histological features), treatment information (type of ET, ribociclib dosage, discontinuation, and interruption), and clinical outcomes (best response to therapy and disease progression). Furthermore, for the purpose of the study, we recorded cutaneous and non-cutaneous adverse reactions, type of cutaneous manifestation, time of onset of dermatological AEs, severity of skin reaction according to the Common Terminology Criteria for Adverse Events (CTCAE—Version 5.0), course, and management. CAEs were considered related to ribociclib according to Naranjo score ≥ 5.^28^ We did not include alopecia among the CAEs, as adnexal reactions are beyond the scope of this work. Identification and management of CAEs were conducted interdisciplinary with a dedicated dermatologist.

The research protocol was approved by the local Ethical Committee (Independent Ethical Committee IRCCS Humanitas Research Hospital, protocol number ONC/OSS-06/2023). Written informed consent for treatment and use of clinical data for scientific purposes was provided by all patients. This study was conducted according to the principles of the Helsinki Declaration.

All patients received ribociclib at the standard initial dose of 600 mg orally once daily, administered 3 weeks on and 1 week off, in combination with ET, either letrozole (2.5 mg per o.s. once daily) or fulvestrant (500 mg intramuscularly on days 1, 14, 28, and then every 28 days) with or without luteinizing hormone–releasing hormone (LHRH) analog, according to menopausal status. All patients were followed up every 4 weeks for the first 3 months and every 4-8 weeks thereafter, or more frequently depending on clinical need. Tumor assessment was performed by computed tomography scan or magnetic resonance imaging according to Response Evaluation Criteria in Solid Tumors (RECIST) version 1.1. Dose reduction and treatment discontinuation were managed as per label indication.

### Study Objectives

The main objective of the study was to determine the incidence of CAEs related to ribociclib in our population. Secondary objectives included the description of types, severity, duration, management, and outcome of ribociclib-induced CAEs and the identification of its potential clinical predictors. Additionally, we aimed to test whether it could be associated with a better survival outcome.

### Statistical Analysis

Clinical data were summarized as frequencies and percentages or as medians and relative ranges. Logistic models were used to test the relationship among potential clinical predictive factors and the occurrence of CAEs. Progression-free survival (PFS), defined as the time from ribociclib first dose until progression or death, was estimated by Kaplan-Meier method and the log-rank test was used to test differences between patients experiencing CAEs to those who did not. All *P*-values were 2 sided, and statistical significance was assumed at *P* ≤ .05, by correcting for multiple tests using Bonferroni. Data analyses were performed using STATA 15 software (StataCorp, 2017, Stata Statistical Software: Release 16, College Station, TX, USA: StataCorp LLC).

## Results

### Patients and Treatment Characteristics

A total of 91 patients with ABC treated with ribociclib were included in the study. Clinical characteristics are summarized in Table 1. The incidence of CAEs due to ribociclib was 14.3% (*n* = 13). All grades neutropenia, hepatobiliary toxicity, and prolonged QT interval were reported in 55.1% (*n* = 43), 11.5% (*n* = 9), and 8.9% (*n* = 7), respectively. In detail, 5 patients who received ribociclib plus fulvestrant and 8 who received ribociclib plus letrozole experienced CAEs. Of these, only 2 patients were premenopausal and received a combination of LHRH analog, ribociclib, and letrozole. None of the concomitant medications had known pharmacological interactions with ribociclib. Three out of 13 patients who experienced CAEs had a personal history of allergy to iodinated contrast media, succinylcholine, and opioids, as well as nickel sulfate.

**Table 1.** Clinical characteristics of patients who experienced CAEs and those who did not.

	CAEs	No CAEs
*N* (%)	13 (14.3)	78 (85.7)
ECOG PS		
0	10 (76.9)	59 (75.6)
1	3 (23.1)	19 (24.4)
Allergies	3 (23.1)	21 (26.9)
History of skin disease	3 (23.1)	0 (0.0)
Recurrent ABC	10 (76.9)	41 (52.6)
De novo ABC	3 (23.1)	37 (47.4)
Luminal A	6 (46.2)	20 (25.6)
Luminal B	7 (53.8)	58 (74.4)
First-line therapy	12 (92.3)	71 (91.1)
HT companion		
Fulvestrant	5 (38.5)	10 (12.8)
Letrozole	6 (46.2)	39 (50.0)
LHRHa + letrozole	2 (15.4)	28 (35.9)
LHRHa + fulvestrant	0 (0.0)	1 (1.3)
Best response		
CR	2 (15.4)	10 (12.8)
PR	6 (46.2)	19 (24.4)
SD	4 (30.8)	43 (55.1)
PD	1 (7.7)	6 (7.7)

Percentage is referred to each subpopulation (patients with or without cutaneous adverse reactions).

Abbreviations: CAEs, cutaneous adverse events; PS, performance status; ABC, advanced breast cancer; HT, hormonal therapy; LHRHa, luteinizing hormone–releasing hormone analog; CR, complete response; PR, partial response; SD, stable disease; PD, progression disease.

### Characteristics and Management of Ribociclib-Related CAEs

Characteristics and management of ribociclib-related CAEs are reported in Table 2. The mean time from the start of treatment to the first detection of CAEs was 3.9 months (range: 0.4-14.4). Most patients experienced severe skin manifestations (8 patients G3, 4 patients G2, and 1 G1). Itch was invariably present in all patients who experienced any type of ribociclib-related CAEs. In 2 of them, pruritus was the exclusive CAEs. Beyond itch, the most frequent reactions included eczematous dermatitis (*n* = 7, 53.8%), followed by maculo-papular reaction (*n* = 2, 15.4%). We also observed isolated cases of urticarial reaction and lichenoid dermatitis.

**Table 2.** Demographic and clinical features of patients with ribociclib-related CAEs.

Patient no.	Age (years)	Known allergies	Associated oncological drugs	Months to CAEs	CAE type, severity grade	Location(s)	Management	CAE relapse, grade	CAE relapse management	Ribociclib response
1	69	Iodinated contrast media	Fulvestrant	0.7	Eczematous dermatitis, G3	H, N, T, UL, LL	Ribociclib interruption and dose reduction (400 mg), methylprednisolone aceponate 0.1% cream, cetirizine 10 mg daily	Yes, G1		SD
2	67	None	Letrozole, denosumab	14.4	Eczematous dermatitis, G3	T, UL	Ribociclib interruption and dose reduction (400 mg), methylprednisolone aceponate 0.1% cream, cetirizine 10 mg daily	Yes, G1		SD
3	69	None	Letrozole	6.1	Pruritus, G2	UL, LL	Ribociclib withdrawal, desloratadine 5 mg daily, switch to palbociclib	No		PR
4	74	None	Fulvestrant	3.0	Maculo-papular reaction, G2	LL	Ribociclib interruption and dose reduction (400 mg)	Yes, G3	Switch to palbociclib	PR
5	72	None	Letrozole	4.1	Lichenoid dermatitis, G1	T, UL, LL	Ribociclib interruption, moisturizer cream	Yes, G3	Ribociclib dose reduction (400mg)	PR
6	70	None	Fulvestrant	3.5	Nummular eczema (eczematous dermatitis), G3	H, N, T, UL, LL	Ribociclib interruption and dose reduction (400 mg), betamethasone dipropionate 0.05% cream	No		SD
7	73	None	Letrozole	0.4	Pompholyx/dyshidrotic eczema (eczematous dermatitis), G3	UL	Ribociclib interruption and dose reduction (400 mg)	No		SD
8	45	Opioids, nickel sulphate	LHRHa, letrozole	4.0	Eczematous dermatitis, G3	T, UL; LL	Ribociclib interruption and dose reduction (400 mg), betamethasone dipropionate 0.05% cream, cetirizine 10 mg daily	Yes, G3	Dupilumab 300 mg q2week	PR
9	75	None	Letrozole	1.2	Eczematous dermatitis, G2	UL	Ribociclib interruption and dose reduction (400 mg)	No		CR
10	65	None	Fulvestrant	2.3	Pruritus, G2	T, UL; LL	Ribociclib interruption	No		CR
11	58	Succinylcholine chloride	Fulvestrant	3.2	Maculo-papular reaction, G3	T, UL; LL	Ribociclib withdrawal			PR
12	46	None	LHRHa, letrozole	5.0	Urticaria, G3	T, UL; LL	Ribociclib interruption and dose reduction (400mg), prednisone 25mg daily, levocetirizine 5mg daily, betamethasone dipropionate 0.05% cream	Yes, G3	Switch to palbociclib	PR
13	67	None	Letrozole	2.6	Eczematous dermatitis, G3	N, UL, LL	Ribociclib interruption and dose reduction (400mg), prednisone 10mg daily, methylprednisolone aceponate 0.1% cream	No		PD

Itch was present in all patients. Severity of ribociclib-related CAEs was graded according to the Common Terminology Criteria for Adverse Events (CTCAE) v. 5.0.

Abbreviations: CAEs, cutaneous adverse events; CR, complete response; H, head; LHRHa, luteinizing hormone–releasing hormone analog; LL, lower limbs; N, neck; PR, partial response; SD, stable disease; T, trunk; UL, upper limbs.

Overall, in our population, 10 patients (76.9%) temporarily interrupted ribociclib treatment and then resumed it at a lower dosage of 400 mg daily. Among these 10 patients, 4 had to reduce the dosage due to both skin-related AEs and other ribociclib-related CAEs, such as neutropenia (3 out of 4) and QT prolongation (1 patient). The first-line treatment of ribociclib-related CAEs included oral antihistamines, moisturizing cream, topical steroids, and/or systemic steroids. Even though these interventions proved effective, 6 patients (46.1%) experienced a recurrence of CAEs. A detailed description of each dermatological entity is reported below.

#### Eczematous Dermatitis

**Figure 1.**
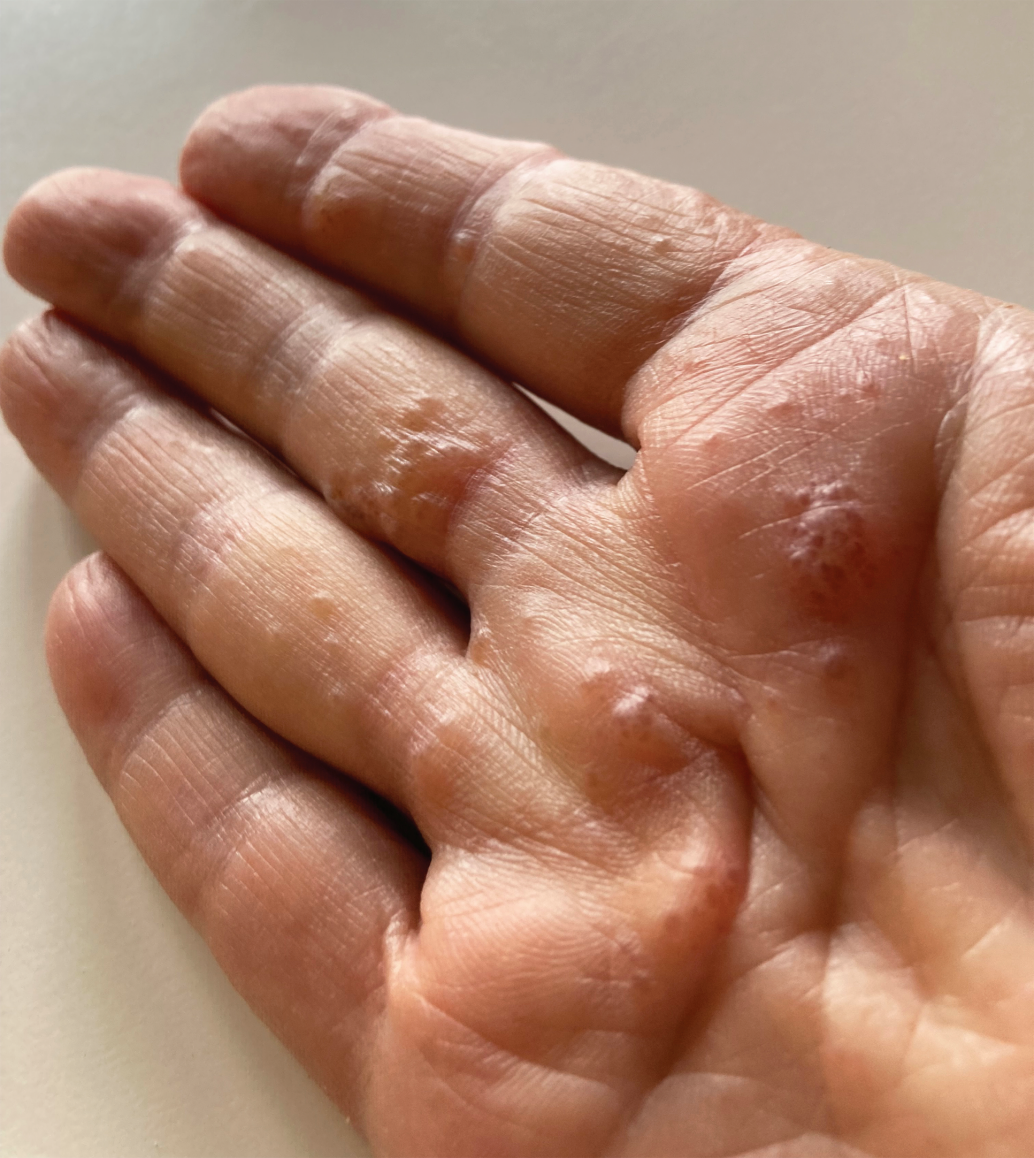
Dyshidrotic eczema (also referred to as pompholyx) of the right hand. This eczematous dermatitis of G3 severity occurred in a 73-year-old woman after the first ribociclib course.

**Figure 2.**
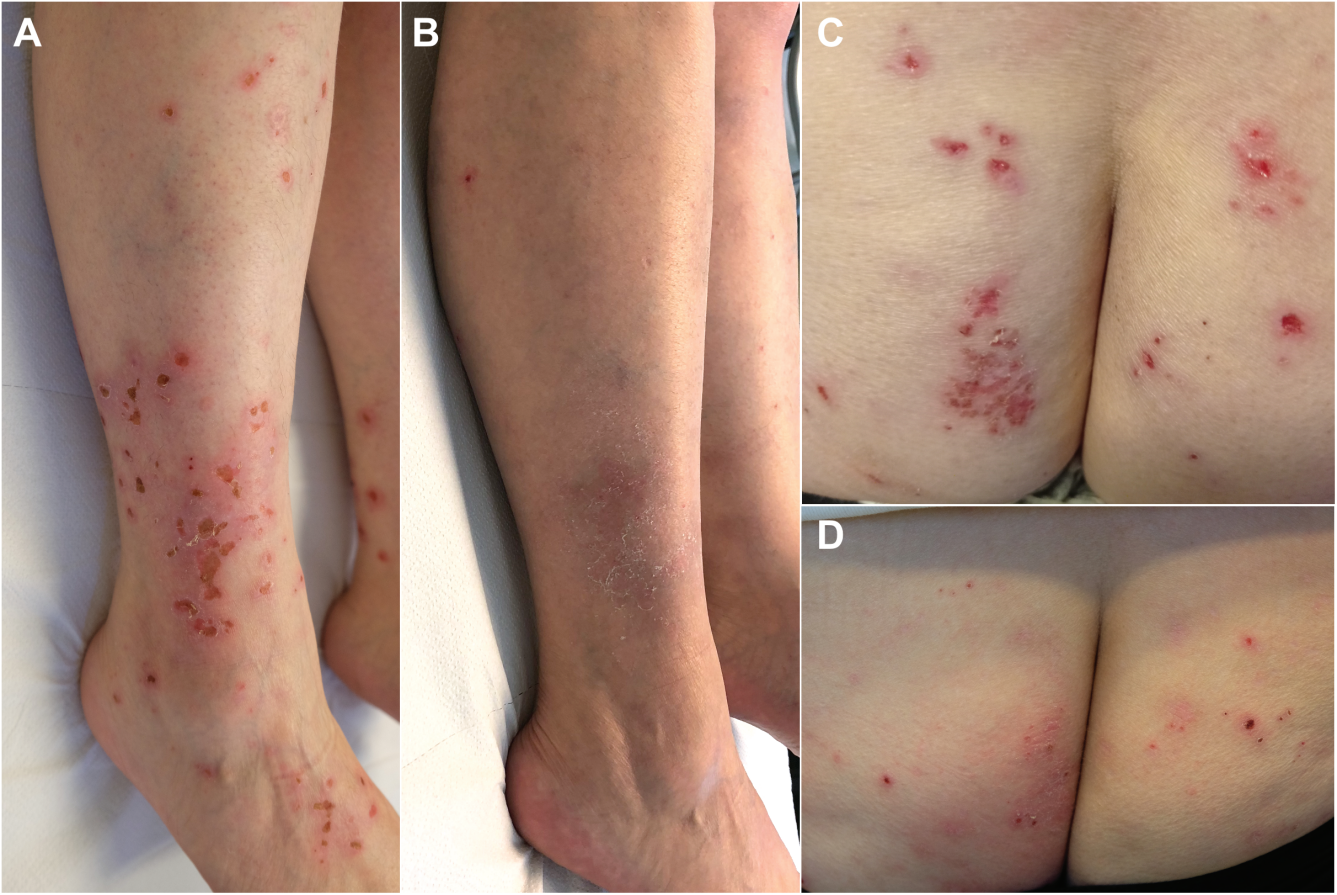
Eczematous G3 dermatitis in a 45-year-old woman with history of allergy to opiods and nickel sulfate, after 4.0 months from the start of ribociclib treatment. Excoriated lesions were widespread on the trunk, upper, and lower limbs (**A, C**). The patient is maintaining almost complete clearance of skin lesions after 18 weeks of treatment with dupilumab 300 mg s.c. every 2 weeks (**B, D**).

The majority of patients (7 of 13; 53.8%) with ribociclib-related CAEs had clinical features consistent with eczema.^29^ Severity was graded as G3 in 6 patients and G2 in 1 patient. Two of the patients with G3 reported a personal history of allergy (iodinated contrast media in one case, opioids and nickel sulfate in the other one). In all patients the lesions were located at least on the upper limbs (Fig. 1); in 10 they also involved the trunk and in 2 the head and neck district. All patients had been initially treated with high-potency topical glucocorticoids and nonsedating oral antihistamines. All patients experiencing eczematous dermatitis during ribociclib therapy required temporary interruption and subsequent ribociclib dose reduction. Eczema relapsed in 3 of 7 patients after reintroduction of ribociclib 400 mg daily, in 2 of them being G1. In one patient, eczema relapsed with G3 severity, and dupilumab 300 mg s.c. every 2 weeks was administered. This patient is currently maintaining partial response after 12 months of treatment, with complete remission of the eczematous dermatitis (Fig. 2), enabling continuation of ribociclib.

#### Maculo-Papular Reaction

Two patients (15.4%) presented with a maculopapular reaction. A 74-year-old patient developed a G2 maculo-papular reaction on her lower limbs and feet. Ribociclib was temporarily interrupted and then resumed at a lower dose (400 mg/day), but a G3 recurrence prompted definitive discontinuation of ribociclib, and switching to palbociclib, with a better CAE profile. The second patient, a 58-year-old woman with a history of succinylcholine chloride allergy, developed a widespread G3 maculopapular reaction that completely regressed upon ribociclib discontinuation; ribociclib was not restarted but maintenance therapy with fulvestrant was continued until progression.

#### Urticarial Reaction

A 46-year-old patient presented G3 urticarial reaction involving the trunk, upper limbs, and lower limbs, approximately 5 months after the first administration of ribociclib. The treatment was stopped and she was given oral prednisone 25 mg daily, levocetirizine 5 mg daily, and betamethasone dipropionate 0.05% cream. The reaction relapsed with G3 severity upon reintroduction of ribociclib, which was therefore switched to palbociclib, with good skin tolerability.

#### Lichenoid Dermatitis

**Figure 3.**
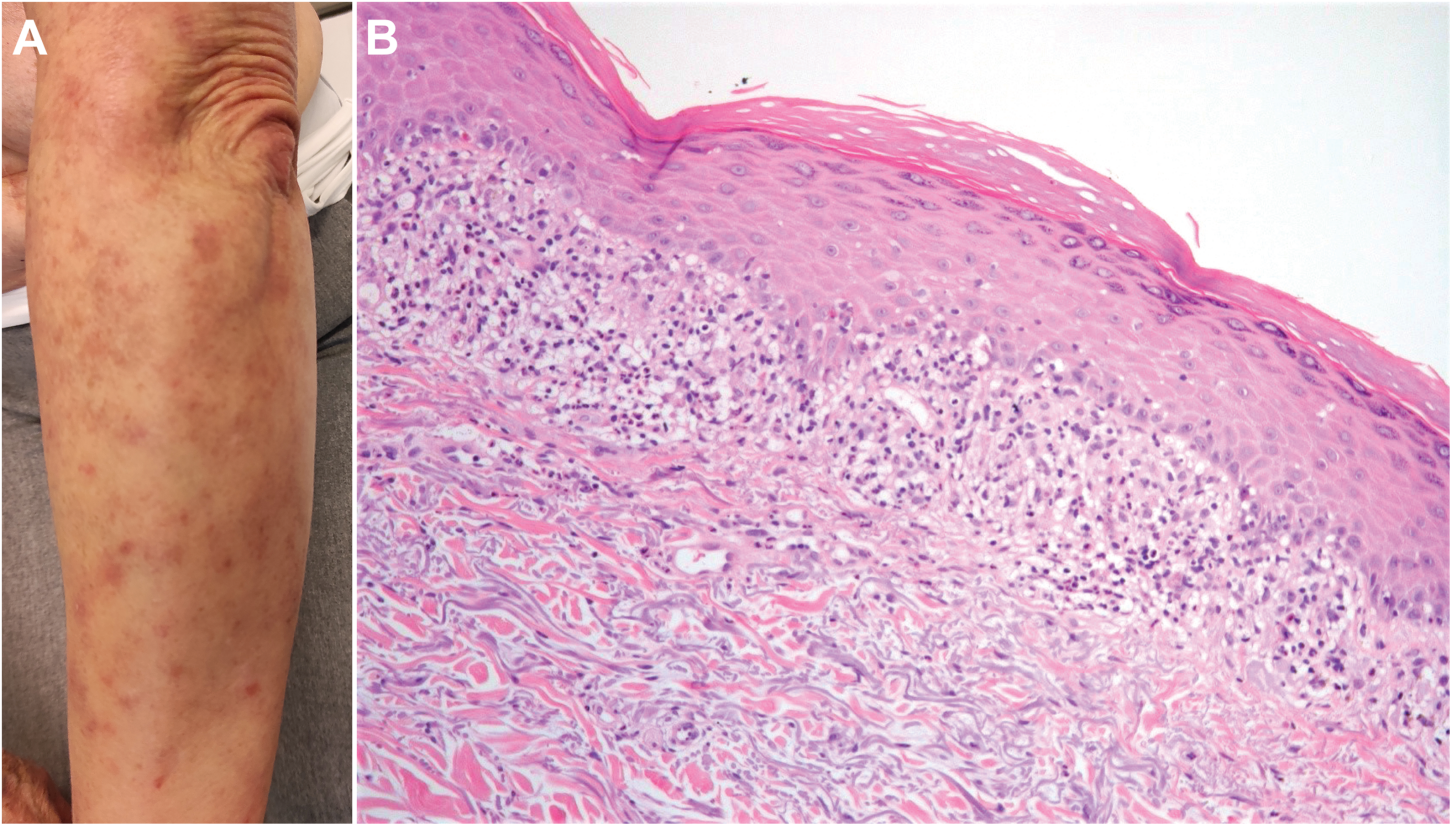
Pruritic macules and papules on the extensor surface of the left forearm in a 72-year-old woman after 4.1 months of ribociclib treatment (**A**). At the skin biopsy, compact hyperkeratosis, hypergranulosis, and a band-like infiltrate of lymphocytes in the superficial dermis were described (hematoxylin and eosin, original magnification ×20; **B**).

A 72-year-old patient developed G1 lichenoid dermatitis involving trunk, upper and lower limbs, 4.1 months after starting ribociclib. In this patient, histopathological examination of a skin biopsy specimen was consistent with the clinical diagnosis of lichenoid dermatitis (Fig. 3). Initial management included topical emollients and delaying the following ribociclib course, with standard dosage. However, the patient experienced worsening of the reaction with G3 eruption during the subsequent ribociclib course, which was therefore reduced to 400 mg daily, with complete resolution of the dermatitis.

#### Pruritus in the Absence of Primary Skin Lesions

A 69-year-old and a 65-year-old woman presented uniquely G2 pruritus, respectively, 6.1 and 2.3 months after the introduction of ribociclib. In the first patient, management required ribociclib withdrawal and oral antihistamine introduction (desloratadine 5 mg daily), while in the second one, there was a total remission of the reaction only by postponing the following cycle. None of them experienced relapse of pruritus, although, in the first patient, ribociclib was switched to palbociclib.

### Clinical Predictors of CAEs and Prognosis

Supplementary Table S1 presents odds ratios (OR) from logistic models that tested potential predictors for CAEs. However, no statistically significant predictor was detected. Luminal A-like subtype, characterized by high-expression levels of estrogen and progesterone receptors (ER and PgR, respectively) and low ki67, showed marginal significance at the univariate logistic regression (*P* = .038). Nevertheless, the value was not significant after a Bonferroni correction, which is recommended when performing multiple tests on the same data.

At a median follow-up of 20 months, the median PFS was 13 months (range, 1-66). Patients who experienced CAEs had a better PFS survival estimate (*P* = .04).

## Discussion

The MONALEESA trials, which led to the approval of ribociclib, reported CAEs as one of the observed side effects. However, the current evidence on specific ribociclib-related CAEs is limited. A systematic review of the literature on CDK 4/6i revealed 13 different adverse skin reaction types related to the use of this class of drugs. Despite alopecia was the most common event, adnexal AEs were not included in the present analysis. Bullous skin reactions represent an important clinical entity that generally caused ribociclib discontinuation. Other events reported in the review were Stevens-Johnson syndrome/toxic epidermal necrolysis, vitiligo-like lesions, erythema dyschromicum perstans, toxic epidermal necrolysis, radiation recall and radiation dermatitis, Henoch-Schonlein purpura, cutaneous leukocytoclastic vasculitis, subacute and chronic cutaneous lupus erythematosus, and histiocytoid Sweet syndrome.^18^

While prior studies have specifically focused on palbociclib-related CAEs,^19^ few studies have been carried out on ribociclib-induced CAEs beyond phase III trials and case reports.^18,22,24-26^

In this study, we found an incidence of ribociclib-induced skin toxicities of 14.3% in a real-world population of 91 patients with HR+/HER2− ABC. The mean time for the development of an adverse skin reaction was 3.9 months after drug initiation. Eczematous dermatitis was the most common cutaneous adverse reaction, followed by maculopapular reaction, urticaria, and lichenoid dermatitis. Itch was invariably present in all patients with ribociclib-related CAEs. The majority of patients reported eczematous changes of moderate severity, mostly manageable through ribociclib interruption, dose reduction, and a multidisciplinary approach to each case. Two patients discontinued therapy due to treatment-related AEs, while 10 patients resumed the same therapy at a lower dosage of 400 mg/day (2 of these 10 patients then switched to palbociclib). Postponing therapy allowed total remission of the reaction in one patient.

**Figure 4.**
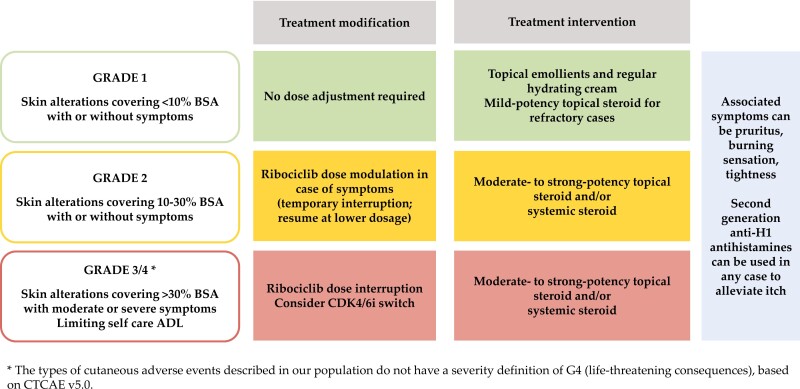
Management of ribociclib-related cutaneous adverse reactions. Abbreviations: BSA, body surface area; ADL, activities of daily living; CDK4/6i, cyclin dependent kinase 4/6 inhibitor; CTCAE, Common Terminology Criteria for Adverse Events.

In addition to considering ribociclib interruption until resolution of the AE (at least in moderate to severe cases), the management of cutaneous adverse reactions included commonly prescribed agents in dermatology. More specifically, for G1 reactions, we recommend topical emollients and regular hydrating cream, while mild steroids can be used in refractory cases. In G2-G3 reactions, topical steroid therapy of moderate to strong potency and/or systemic steroids should be considered. Antihistamines may be added to alleviate itch in any grade. In all cases, it is crucial to seek early dermatology consultation to ensure greater diagnostic accuracy and appropriate management, including targeted therapy, according to the clinical presentation. In moderate to severe (G2-G3) skin reactions, it is always a good practice to consider ribociclib suspension until the resolution of the AE. Figure 4 depicts a simplified representation of possible management of ribociclib-induced cutaneous adverse reactions.

We did not detect any clinical predictor of CAEs. Nonetheless, the identification of biomarkers through clinical research is a challenge that should be taken up by future studies, as this can lead to their successful implementation in routine clinical practice. Such biomarkers, together with dermatological consultation, could be helpful to reduce or even to prevent cutaneous adverse reactions.

A PFS analysis showed a better curve for patients experiencing CAEs (*P* = .04). This is consistent with previous studies showing better outcomes for patients who develop CAEs in response to antiepidermal growth factor receptor-targeted agents in different tumor types, including non–small cell lung cancer and colorectal cancer (CRC). For instance, in lung cancer patients treated with erlotinib as a single agent or with cetuximab plus chemotherapy, the early development of CAEs has been associated with remarkably better PFS and OS outcome.^30,31^ A similar relationship between CAEs and clinical outcomes was reported in studies of cetuximab and panitumumab in CRC.^27^ Nonetheless, the clinical significance of this association between CAEs and treatment efficacy remains to be confirmed in larger studies.

Furthermore, the possible etiology underlying the development of cutaneous adverse reactions needs further investigation. A study conducted on a rat model identifies the suppression of adenosine 5ʹ-triphosphate (ATP) as a possible reason for skin damage, based on the ribociclib mechanism that inhibits the activity of CDK4/6 by competitively binding to ATP binding sites. The study speculated that ATP may be useful in the treatment of ribociclib-induced skin damage, but this evidence needs to be validated in humans.^32^

Our study should be interpreted in light of 2 main limitations. First, the monocentric nature of our study might raise concerns in terms of sample size. Second, results from our sample indicate that skin manifestations were severe in most cases (8 patients G3, 4 patients G2, and 1 G1), contrary to what was reported in pivotal trials. This is possibly due to the underrepresentation of patients with G1 CAEs in our retrospective study. As a matter of fact, these could be underreported during the oncological visits, since patients with a G1 CAE tended to avoid reporting it due to its easy management and rapid resolution.

## Conclusion

Our research indicates that the interdisciplinary management of patients with ABC is a crucial aspect in the early management of treatment-induced toxicities. An integrated approach involving ribociclib dose modulation and specific dermatological interventions prevented permanent discontinuation of ribociclib in the majority of patients (69.2%). By incorporating this approach into routine care, clinicians can enhance treatment adherence and optimize patient outcomes in this specific patient population. In fact, continuing treatment with CDK4/6i while maintaining a high quality of life represents an actual challenge for the treatment of HR+/HER2− ABC.

## Supplementary Material

oyae004_suppl_Supplementary_Tables_S1

## Data Availability

The data presented in this study are available on reasonable request from the corresponding author.
