# Supplementary material for: Ribociclib-Induced Cutaneous Adverse Events in Metastatic HR+/HER2− Breast Cancer: Incidence, Multidisciplinary Management, and Prognostic Implication
Source: Oncologist. 2024 Jan 18;29(6):484–92. doi: 10.1093/oncolo/oyae004 (PMC11145009; doi:10.1093/oncolo/oyae004)
Supplement: oyae004_suppl_Supplementary_Tables_S1 [file oyae004_suppl_supplementary_tables_s1.docx]

**Supplementary Table 1**. Odds ratios from logistic models testing the relationship of potential predictors on our main outcome of interest that is skin toxicity.

| Dependent Variable: Skin Adverse Event | | | | | | | |
| --- | --- | --- | --- | --- | --- | --- | --- |
|  |  | (1) | (2) | (3) | (4) | (5) | (6) |
|  |  | *N = 91* | *N = 91* | *N = 91* | *N = 91* | *N = 91* | *N = 91* |
| Menopausal status |  | 0.45 |  |  |  |  | 0.62 |
|  |  | (0.313) |  |  |  |  | (0.475) |
| ECOG PS |  |  | 0.87 |  |  |  | 0.56 |
|  |  |  | (0.533) |  |  |  | (0.375) |
| LuminalA |  |  |  | 3.53* |  |  | 3.40 |
|  |  |  |  | (2.145) |  |  | (2.164) |
| Comorbidities |  |  |  |  | 1.60 |  | 1.95 |
|  |  |  |  |  | (0.938) |  | (1.301) |
| Allergies |  |  |  |  |  | 0.21 | 0.19 |
|  |  |  |  |  |  | (0.219) | (0.213) |
| Constant |  | 0.23*** | 0.19 | 0.10*** | 0.14*** | 0.23*** | 0.12*** |
|  |  | (0.077) | (0.060) | (0.046) | (0.062) | (0.071) | (0.081) |
| **** p-value < 0.001; ** p-value < 0.01; * p-value < 0.05* | | |  |  |  |  |  |
